# The Challenge of the Pathogenesis of Parkinson's Disease: Is Autoimmunity the Culprit?

**DOI:** 10.3389/fimmu.2018.02047

**Published:** 2018-09-27

**Authors:** Tianfang Jiang, Gen Li, Jun Xu, Shane Gao, Xu Chen

**Affiliations:** ^1^Department of Neurology, Shanghai Eighth People's Hospital Affiliated to Jiang Su University, Shanghai, China; ^2^Department of Neurology & Institute of Neurology, Rui Jin Hospital Affiliated to Shanghai Jiao Tong University School of Medicine, Shanghai, China; ^3^East Hospital, Tong Ji University School of Medicine, Shanghai, China

**Keywords:** autoimmunity, Parkinson's disease, α-synuclein, autoimmune diseases, neuroimmunology

## Abstract

The role of autoimmunity in Parkinson's disease (PD), as one of the most popular research subjects, has been intensively investigated in recent years. Although the ultimate cause of PD is unknown, one major area of interest remains identifying new therapeutic targets and options for patients suffering from PD. Herein, we present a comprehensive review of the impacts of autoimmunity in neurodegenerative diseases, especially PD, and we have composed a logical argument to substantiate that autoimmunity is actively involved in the pathogenesis of PD through several proteins, including α-synuclein, DJ-1, PINK1, and Parkin, as well as immune cells, such as dendritic cells, microglia, T cells, and B cells. Furthermore, a detailed analysis of the relevance of autoimmunity to the clinical symptoms of PD provides strong evidence for the close correlation of autoimmunity with PD. In addition, the previously identified relationships between other autoimmune diseases and PD help us to better understand the disease pattern, laying the foundation for new therapeutic solutions to PD. In summary, this review aims to integrate and present currently available data to clarify the pathogenesis of PD and discuss some controversial but innovative research perspectives on the involvement of autoimmunity in PD, as well as possible novel diagnostic methods and treatments based on autoimmunity targets.

## Introduction

The consensus is that under normal physiological conditions, the whole immune system fights against foreign antigens but not self-aggressors. Unfortunately, long-standing studies have revealed that immunological destruction may incite organisms to attack the self-antigens of cells or tissues, referred to as autoimmunity (1, 2). Failure to maintain the self-tolerance of lymphocytes is a fundamental explanation for the onset of autoimmune diseases. The pathogenesis of autoimmunity has been explored for many decades, and several relevant mechanisms have been confirmed to cause autoimmune diseases, which are summarized as follows: (1) genetic alterations in pattern recognition receptors (PRRs); (2) cross-reaction of immune cells with self-antigens (also called molecular mimicry); (3) epitope spreading or drifting; and (4) dysfunction of T cells and B cells. Specifically, Chastain and Schie have shown that genetic alterations in PRRs could increase the sensitivity threshold against harmless self-antigens. They also demonstrated that autoimmunity could result from cross-reactivity between a host cell receptor and the antibody induced by the antigenic epitope of an antiviral agent (3, 4). Qiao et al. attributed the occurrence and development of an autoimmune disease to an imbalance between regulatory T cells (Tregs) plus suppressive cytokines and effector T cells plus pro-inflammatory cytokines (5). The powerful immune suppressive capacity of Tregs and their secreted cytokines could suppress not only effector T cells but also other immune cells, such as B cells and dendritic cells (DCs). Meanwhile, it has also been demonstrated that the assistance of CD4^+^ cells (also known as helper T cells) is pivotal for the autoantibody response of B cells driven by autoantigens, which can also improve the outcome of immune reactions initiated by various antigen-presenting cells (APCs) as a secondary response to antigens (6, 7). This review mainly elaborates on how inappropriate immune responses in the central nervous system (CNS) contribute to the pathogenesis of a broad range of neurodegenerative disorders including but not be limited to Parkinson's disease (PD).

## Autoimmunity in neurodegenerative diseases and its relevance to PD

The immune system always exerts intricate and reciprocal effects on the nervous system. Previous research considered brain cells safe from attack by the immune system because most neurons do not express antigens, which are markers specifically recognized by antibodies. Nevertheless, increasing data have indicated that autoimmunity causes neuronal demyelination, axonal damage, synaptic loss and further neurodegeneration (8). In fact, the CNS usually suffers from a chronic autoimmune attack. According to Kawai and Akira, inflammation is one of the first and most prominent events in this chronic process (9), which can last a decade or two, followed by the accumulation of neuronal injury, eventually resulting in irreversible neurodegeneration. When autoimmunity begins, some harmful cytokines are released, some of which further recruit immune cells to continuously attack neurons and nerve fibers (10–12). Multiple sclerosis is a torturous autoimmunity-related CNS disease with typical pathological variances that are usually clinically marked by oligoclonal bands and/or an increased immunoglobulin G index (13). As mentioned above, even though the damage associated with acute inflammatory lesions occurs first, the subsequent autoimmunity-induced neurodegeneration is linked with the progressive development of disability (14, 15). Overall, neurodegenerative diseases are irreversible, and the related deterioration might be due to the chronic, long-lasting, and autoimmunity-induced pathology transformation. Meanwhile, advanced age, one of the main risk factors of both neurodegeneration and autoimmune disease, is characterized by an erosion of tolerance and increased reactivity to self-antigens (16–19). As such, it is assumed that PD, as one of the most common neurodegenerative disorders ranking after Alzheimer's disease (AD), is also likely to be an autoimmune disease.

**Figure 1:**
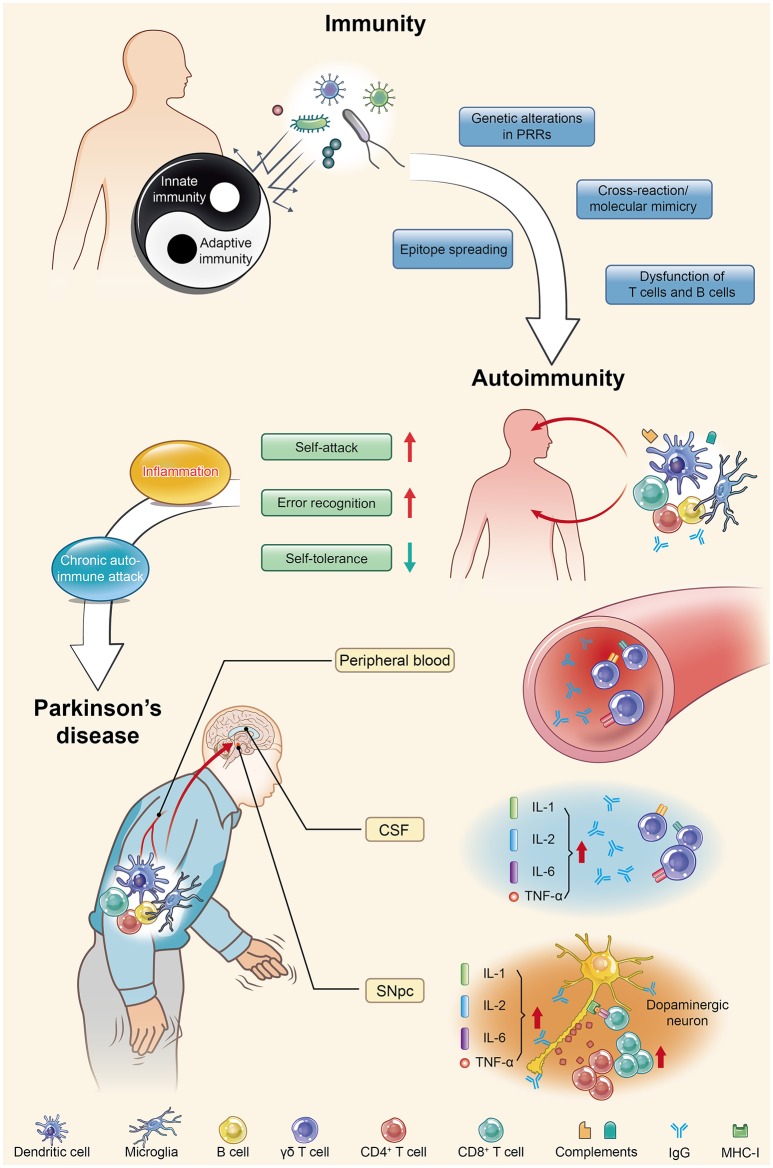
Parkinson's disease (PD) is actually an autoimmune disease. Autoimmunity occurs when immune homeostasis is broken by several main mechanisms shown in this figure, which directly result in an increase in error recognition and self-attack and a decrease in self-tolerance to autoantigens. Regarding PD, chronic autoimmune attack is not only its pathogenesis but also always involved throughout the entire disease process. Inflammation is the first step of this attack, with the subsequent participation of various immune cells and immunoglobulins they produce, ultimately leading to the death of dopaminergic neurons. PRRs, pattern recognition receptors; CSF, cerebrospinal fluid; SNpc, substantia nigra pars compacta; IL, interleukin; TNF, tumor necrosis factor.

The pathogenesis, diagnosis and treatment of PD have received increasing interest due to the increasing morbidity and mortality, enervating features, irreversibility, and early-onset tendency of the disease. In terms of the mechanism of the death of dopaminergic neurons (DNs), no unanimous conclusion can yet be drawn. A growing number of published studies using cell culture systems and preclinical animal models have provided evidence for a role of the immune system in the etiology of PD (20–22). Some researchers had already begun to focus on the relationship between PD and autoimmunity as early in 1989; however, due to sample size limitations and immature experimental technology, they did not obtain reliable data showing a significant correlation between PD and autoimmunity (23). After nearly three decades, a series of research results have demonstrated that both the innate and adaptive immune systems are activated in PD. Significant increases in innate immune factors, including interleukin (IL)-1, IL-2, and IL-6 and tumor necrosis factor (TNF)-α, have been detected within the substantia nigra pars compacta (SNpc) and cerebrospinal fluid (CSF) of PD patients (24, 25), and γδ T cells, the first line of defense, have also been found to be elevated within the peripheral blood and CSF (26). For specific recognition, human catecholaminergic SNpc neurons express major histocompatibility complex I (MHC-I), which enables them to present autoantigens and be more susceptible to T cell-mediated cytotoxic attack (27). Increased levels of specific immunoglobulins in the peripheral blood and CSF of PD patients have further suggested that humoral autoimmunity is involved in the pathogenesis of PD (28–30). Additionally it became more convincing that post-mortem studies of PD brain tissue showed both CD4^+^ and CD8^+^ T cells in close proximity to DNs within the SNpc at levels 10-fold higher than in the control group (31). Analysis of the correlation between immunity and PD has demonstrated that immunoglobulin G (IgG) binds to DNs in PD (32). Moreover, an increase in CD8^+^ T cells and a decrease in Tregs within the peripheral T lymphocyte populations of PD patients (33) indicated the downregulation of self-tolerance and upregulation of error recognition and self-attack, further corroborating the potential involvement of autoimmunity in PD progression. All of these reliable experimental data indicate that autoimmunity might play a key role in PD development. More in-depth studies are urgently needed to prove that autoimmunity is the main cause of PD and to explain the mechanism underlying the injury and selective loss of DNs. Autoimmunity contributes to the pathogenesis of PD in a multifactorial manner involving α-synuclein (α-syn) and immune cells (e.g., microglia and DCs) and the mutation of many genes (e.g., *PINK1, Parkin*, and *DJ-1*). These contributions produce varied and unique corresponding pathomechanisms and clinical features, which will be discussed at length in the following sections in sequence (Figure 1).

## Genetic regulation of autoimmunity in PD

Some autoimmune diseases are frequently familial, while other autoimmune diseases are sporadic (34). Despite the proven genetic associations among distinct autoimmune diseases, much of the heritability remains unaccountable (35). Scientists have assumed that PD-related genes might be the key regulatory factors engaging the autoimmune system and their dysfunction would overshoot immunity either by lost tolerance or increased sensitivity thresholds to self-antigens (36). Undoubtedly, certain genes are closely related to PD, with two inheritance modes: autosomal dominant and autosomal recessive (37). Three genes, *PINK1, Parkin*, and *DJ-1*, are closely related to autosomal recessive genetics in early-onset PD. Meanwhile, mutations in *PINK1* and *Parkin*, which encode a mitochondrially targeted protein kinase and an E3 ubiquitin ligase, respectively, have been found in both familial and sporadic PD (38, 39). It was once believed that the dysfunction of these two genes would cause the failure to maintain normal mitochondrial function, leading to the loss of DNs and ultimately causing PD (39, 40). While in the past few years, it has been shown that *PINK1* and *Parkin*-related immune system disorders are indeed responsible for the upstream mechanism of mitochondrial aberrations. *PINK1*, a kinase stabilized at the surface of mitochondria, phosphorylates both ubiquitin and *Parkin* (41, 42). The reduced ability of *PINK1*^−/−^ CD4^+^ T cells to suppress bystander T cell proliferation indicate that this pathological state could result in reduced immuno-surveillance or activated autoimmunity during PD progression (43). In addition, it has been reported that the loss of *PINK1*/*Parkin*-dependent mitochondrial quality control triggers a series of physiological events related to PD, including the abnormal initiation of innate immunity (44). The lack of *PINK1* and *Parkin* has been confirmed to induce high levels of mitochondrial antigen presentation (MitAP) MHC-I molecules in both macrophages and DCs, as well as accelerating the formation of mitochondria-derived vesicles (MDVs) on which MitAP depends both *in vitro* and *in vivo* (45). Data have also shown that *Parkin*^−/−^ DNs with MitAP activation are recognized by established mitochondria antigen-specific T cells, accompanied by cytotoxic responses, including microglial activation and local inflammation, as well as a significant contribution of the immune system in the etiology of PD (34, 46). During the process of infection or inflammation, the presence of a lymphatic system in the CNS could facilitate the transportation of immune cells into the brain, subsequently destroying DNs expressing mitochondrial antigens on their surface. In other words, under these circumstances, mitochondrial antigen-expressing DNs are much more “visible” to autoimmunity (34, 47). As previously stated, elucidating the abnormal function of T cells in the absence of *PINK1* and/or *Parkin* may also help to unravel the role of autoimmunity in PD. Therefore, further investigations of T cell function in *PINK1* and/or *Parkin* mutation carriers are needed.

In addition to these observations, *DJ-1* (Parkinson's disease protein 7, *PARK7*) has also been reported to affect the development of natural Tregs (nTregs) and induced Tregs (iTregs, previously known as suppressor T cells). Mature Tregs with normal function, which modulate not only adaptive immunity but also innate immunity, are pivotal for maintaining thymic function, peripheral immune self-tolerance and immune system homeostasis. nTregs are generated in the thymus, while iTregs are derived from naïve CD4^+^ T cells encountering antigens in the peripheral organs. Both cell types are generally immunosuppressive through the suppression or downregulation of effector T cell proliferation (48). Their “self-check” function successfully prevents excessive effector cell reactions. On the other hand, the abnormal proliferation of both types of Tregs leads to the failure of self-/non-self-discrimination, resulting in autoimmune disease (49). Evidence reported by Singh et al. has demonstrated that *DJ-1*, one of the most classical key players responsible for PD pathogenesis, is strongly linked with neuroimmunology and multiple autoimmune responses in PD (50). In addition, *DJ-1*-deficient animal models have shown compromised iTreg induction, cell cycle progression, and cell survival and proliferation. *DJ-1*^−/−^ iTregs are more proliferative, more susceptible to cell death signals and deficient in cell division compared with wild type counterparts, as analyzed by flow cytometry and Western blotting.

In conclusion, these discoveries provide a new perspective on the relationship between gene regulation and neuroimmunology. Consistent with previous reports, *PINK1, Parkin* and *DJ-1*, which have been cited as the three musketeers of neuroprotection (51), are beneficial to mammalian organisms. However, deficiency of these genes leads to a failure to maintain normal neuron function and prevent oxidative stress and inflammation damage in PD, which has also been confirmed by our previous studies (52, 53). Similarly, a failure to maintain the homeostatic immune system leads to a hyperactive autoimmune state and accelerates disease progression.

## Pathogenic protein function in autoimmunity-associated PD

α-Syn, a small synaptic protein and the primary component of Lewy bodies, if incorrectly modified or misfolded, can form soluble or insoluble aggregates and act as the neuropathological hallmark in the brain of patients with either sporadic or familial PD (54). α-Syn plays a leading role in the initiation and progression of Parkinson-like neurodegeneration because it can induce high neurotoxicity by diverse pathways, such as inflammation, oxidative stress and autophagy abnormalities (54, 55). The neurotoxicity of α-syn is largely attributed to its soluble or insoluble aggregates of oligomers or polymers, which are found throughout the SNpc in PD but are also found in other neurons. The hypothesis that α-syn is involved in the autoimmune process driving PD has been constantly and widely debated. Prior experimental evidence in favor of α-syn as a self-antigen in PD is based on data reported by Benner et al., who found that effector T cells immunized by a self-antigen, nitrated-α-syn (typical neuropathology of PD), could exacerbate neuroinflammation and augment the neurodegeneration of SNpc DNs in an 1-methyl-4-phenyl-1,2,3,6-tetrahydropyridine (MPTP) mouse model (56). This finding indicates that nitrated-α-syn, as a self-protein and biomarker for the clinical diagnosis of PD, which was detected readily in cervical lymph nodes from MPTP-intoxicated mice, might break down immunological tolerance and induce the autoimmune responses that exacerbate the pathobiology of PD. This report led to an alternative theory that α-syn causes PD by triggering the immune system to attack the brain. Additionally, Cao et al. injected adeno-associated virus overexpressing α-syn into the SNpc of Fc-γ receptor^−/−^ mice via a stereotaxic method and detected attenuated microglial activation and reduced dopaminergic neurodegeneration compared with non-injected controls (57). Over-abundance of α-Syn lead to the expression of a specific antigen, which further induces IgG generation. The Fc-γ receptor is expressed on the cell membrane of microglia, which binds IgG and triggers signal transduction events leading to microglial activation that eventually injures neurons in the SNpc. Therefore, α-syn is important for inducing an autoimmune response that leads to neurodegeneration. Upon further analysis, views from Heather's team have emphasized that in addition to nitration, another post-translational modification of α-Syn in PD, such as phosphorylation at serine 129 (S129), affects the toxicity, oligomerization, and immunogenicity of α-syn itself (58). Casein kinase-2 and G-protein-coupled receptor are two main kinases which influence the phosphorylation of α-syn (59, 60). Circumstance poisons such as MPTP and paraquat can also cause S129 of α-syn (61). Approximately 90% of α-syn in Lewy bodies is phosphorylated at S129 in PD in the brain, while it is relatively rare in human normal brain tissue (~4%). Thus, researchers have speculated that the epitope of α-syn might not exist in the thymus when facing negative selection and would be erroneously recognized as a foreign antigen (62). In addition to the above findings and analysis, Li and Games and their colleagues found another way to change the antigenicity of α-syn both *in vitro* and *in vivo*. They passively immunized mice using α-syn antibodies designed to bind the gene's C-terminal fragments and successfully observed decreased α-syn aggregation, reduced DN loss, and alleviated movement disorder in the α-syn model of PD (63, 64). It could be concluded that the C-terminal truncation mutant of α-syn, identified in Lewy bodies and brain tissue with PD, possibly produces new antigens induced by altered α-syn processing.

As discussed above, molecular mimicry and cross immunoreactions are two of the primary mechanisms through which autoimmunity is triggered. Molecular mimicry between herpes simplex virus 1 (HSV1) and human α-syn was detected in PD patients in 2016. HSV1 infection could enhance the development of autoimmunity because autoreactive antibodies induced by HSV1 have the same response to the human α-syn homologous peptide bound to the membrane of DNs and lead to DN destruction (65). These results also support the assumption that α-syn participates in autoimmunity involved in the pathological progression of PD.

According to previous reports, MHC proteins are present on SNpc DNs and norepinephrine neurons in the locus coeruleus, and in the presence of the appropriate antigen and CD8^+^ T cells (also known as cytotoxic T cells), MHC-I expressing SNpc murine neurons are more easily destroyed, suggesting that antigenic epitopes could activate CD8^+^ T cells involved in the autoimmune response and cell death (27). In June 2017, Sulzer et al. concentrated on the characteristics of α-syn and tested whether it could be a target of T cells as a potential self-antigen (66). They detected the immune responses of peripheral blood mononuclear cells from 67 PD patients and 36 healthy controls that were exposed to a set of α-syn-derived peptides. It has been shown that the small stretches of α-syn around the Y39 and S129 phosphorylation regions successfully trigger the T cell response. Furthermore, the specific sets of T cells that respond to α-syn epitopes have also been identified to be mostly CD4^+^ and partly CD8^+^ T cells. This information could greatly benefit clinical diagnosis and treatment not only because T cell responsiveness might be a new biomarker for identifying individuals at risk or in the early stages of PD but also because of the potential for strategies for inhibiting the immune reaction or elevating the threshold of recognizing self-antigens, such as α-syn, as an attractive and promising therapeutic target in PD. However, there is still much more exploration to be done, as it is not yet clear if the autoimmune response is the initiator or an important pathogenic component of PD; in either case, it cannot be underestimated. Sulzer's team plans to block the autoimmune response in PD, e.g., by deleting certain T cell subpopulations, B cells or MHC, in an attempt to determine whether this will halt progression of the disease.

## Immune cells and autoimmunity in PD

To date, numerous immune cells have been shown to be responsible for driving PD progression. DCs and microglia are two types of mammalian immune cells that act as the first and main forms of active immune defense in the CNS (67). They act first as APCs and then activate T cells to initiate the immune system to identify and attack extrinsic antigens. In essence, these immune cells lie at the intersection of the immune response and the neurodegenerative process—two primary aspects of CNS autoimmune disorders. DCs, the famous APCs (also known as accessory cells), serve as messengers between the innate and adaptive immune systems and can induce and even maintain self-tolerance (68). It is the differentiation/maturation rather than the haematopoietic origin or subset classification of DCs that determines their tolerogenic or immunogenic functions. Immature DCs can inhibit alloantigen-specific T cell responses to reverse autoimmune diseases in murine models but simultaneously induce antigen-specific T cell tolerance (69). The maturation of DCs into professional APCs via the upregulation of MHC expression enables DCs to capture antigens successfully (70). Based on these phenomena, Platt et al. proposed a theory called “regulatory mechanisms by DCs” for immune responses against self-antigens. They concluded that the failure of DCs to control T cells via Treg differentiation and effector T cell clonal deletion leads to a direct attack on self-antigen-harboring target cells (71).

The progressive loss of neuromelanin (NM)-containing DNs in the SNpc is one of the predominant features of PD. Once produced by dopamine and norepinephrine via an interaction with cysteine as the inevitable by-product of aging (72), NM (the pigment) is no longer merely a spectator but an autoantigen released from dead DNs that stimulates the maturation and functional activation of DCs though being phagocytized by DCs, and then triggers an adaptive autoimmune response and finally leads to microglial activation, which enhances this autoimmunity via positive feedback (73). Subsequently, these mature DCs migrate from the CNS to cervical lymph nodes, resulting in the presentation of NM to naïve T and B cells in a highly immunogenic context (74). This autoimmune response might eventually lead to the death of NM-rich neurons in PD. Therefore, Oberlander and his colleagues inferred that NM is a potential target structure during autoimmune attack on DNs. This conclusion was later supported in 2009, as a relatively higher level of anti-NM antibodies was detected in the sera of PD patients (28). Consistently, a complement factor named C1q, which has been confirmed to be involved in the classical complement pathway and recognize antigen-bound IgG and IgM, was found to localize on the surface of extracellular NM in the brain of post-mortem PD patients (75). These data highlight the conclusion that NM is a potential target during the autoimmune-based pathogenesis of PD. Although DCs rarely exist in the healthy human brain, myeloid-derived DCs can still infiltrate the brain tissue during the process of neuroinflammation (76, 77). The exact mechanism by which NM activates DCs is by its peptide or lipid components, not by the dopamine melamine backbone, because DC maturation is due to the oxidized lipophosphatidylcholine (LPC) found in low-density lipoproteins (LDLs) (74). This point of view is supported by the increased lipid peroxidation in the SNpc detected in post-mortem PD patients.

**Figure 2:**
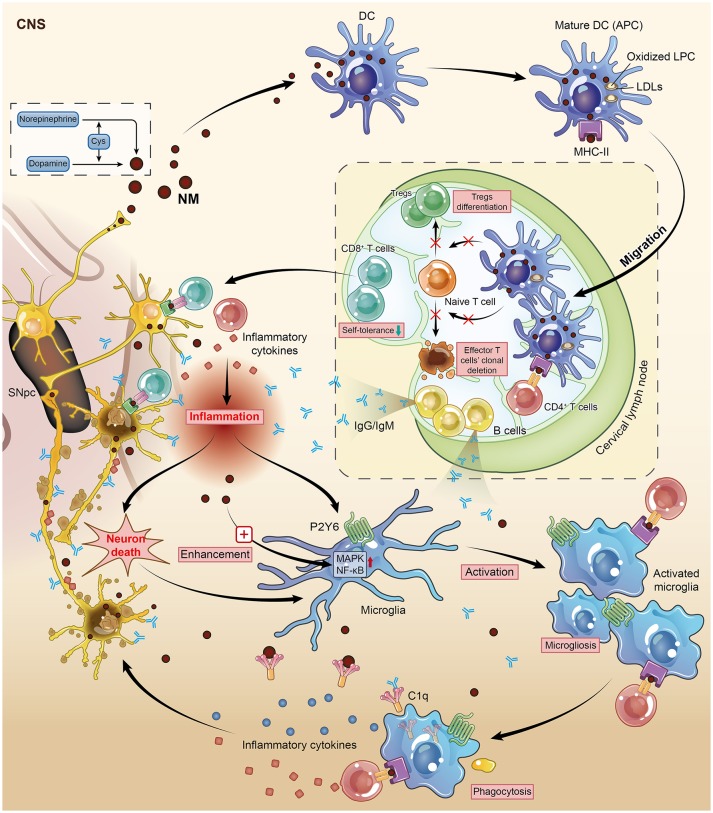
Neuromelanin (NM) is one of the potential targets during the autoimmune-based pathogenesis of PD. This figure illustrates vividly how DCs and microglia, two kinds of mammalian immune cells, interact with each other and identify NM-rich cells as the object of autoimmune attack on DNs. Mature DCs migrate from the CNS to cervical lymph nodes, resulting in the presentation of NM to naïve T and B cells in a highly immunogenic context. This auto-aggressive loop initiated by DCs along with NM is enhanced and amplified by microglial activation. DC, dendritic cell; DN, dopaminergic neuron; CNS, central nervous system; APC, antigen-presenting cell; Cys, cysteine; Tregs, regulatory T cells.

In the context of autoimmune disorder-induced PD, the resulting antigens presented by microglia could promote self-antigen recognition by T cells, thus contributing to neuronal damage. The upregulation of MHC-II on microglia allowed microglia to present self-antigens to autoreactive T cells (78). This auto-aggressive loop initiated by DCs along with NM would be enhanced and amplified by microglial activation. Wilms et al. investigated the effects of NM on the release of neurotoxic mediators and the underlying signaling pathways through microglial culture in rats. NM augmented microglial activation by manipulating two signaling pathways, the p38 mitogen-activated protein kinase (MAPK) and nuclear factor kappa B (NF-κB) pathways (79). Similarly, NM injection into the rat SNpc induced microgliosis and the loss of tyrosine hydroxylase neurons *in vivo*, suggesting a close relationship between microglia and NM-associated DN degeneration in PD (80). As previously mentioned, inflammation acts as the first link between autoimmunity and its subsequent chronic damage, and our findings have suggested that the purinergic receptor P2Y6 mainly contributes to the activation and later phagocytosis of microglia in the CNS, resulting in an outbreak of inflammatory cytokines in the immune system (81). Hence, microglial activation is a downstream event in which microglia present an antigen (like NM) to DC-primed infiltrating T cells to direct the autoimmune response. Overall, DCs and microglia orchestrate the autoimmune response by executing different but cooperative functions during an autoimmune response (Figure 2).

## Clinical features and autoimmunity in PD

**Figure 3:**
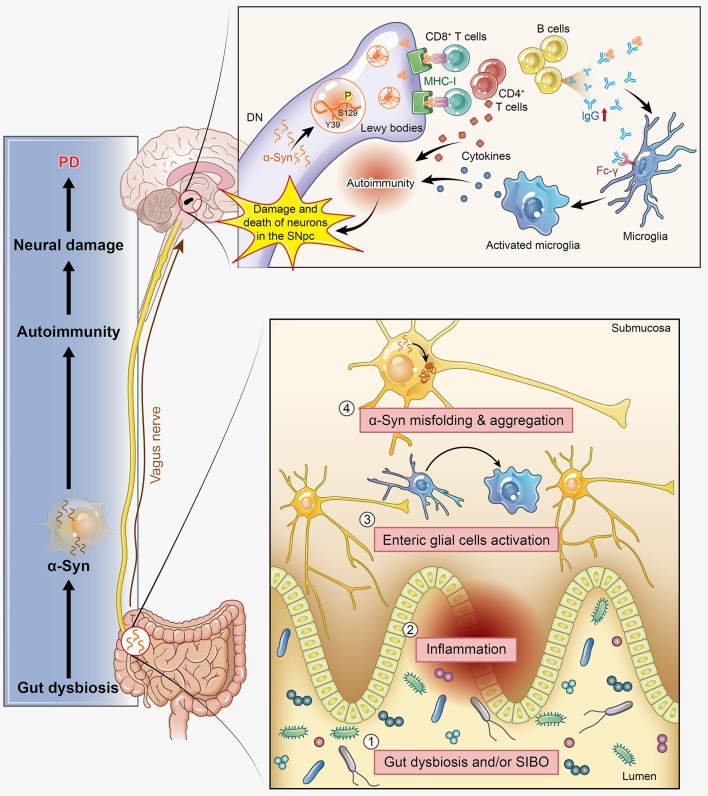
α-Synuclein (α-syn) participates in autoimmunity and is involved in the pathological progression of PD. α-Syn, as the main disease-causing protein, first appears in the gut and is related to gut dysbiosis, which disturbs the intestinal immune system, leading to one of the main non-motor symptoms of PD: constipation. Then, this protein transmits to the dorsal motor nucleus through the vagus nerve and acts as a self-antigen targeted by effector T cells, B cells, and microglia. This autoimmunity attack finally results in the damage and death of DNs. SIBO, small intestinal bacterial overgrowth.

Dyskinesia, rest tremor, muscular rigidity, and gait disorder are the main motor symptoms of PD, while constipation, depression, hyposmia, and somnipathy are the main non-motor symptoms in a few PD patients. These symptoms indicate that there is much to translate from basic bench research into clinical treatment. Thus, the relationship between PD patients' clinical features and autoimmunity is also one of our interests in this review. There are three major, clinically relevant forms of PD: (1) tremor-dominant form; (2) rigidity-dominant form; and (3) gait difficulty form (82). Many clinical studies have provided solid evidence that autoimmunity participates in the pathogenesis of PD. Elevated serum levels of anti-α-syn antibodies have been found to be associated with familial variants of PD (29), and increased anti-GM1-ganglioside antibody levels have been detected in the tremor-dominant form of PD (83). As such, Benkler et al. analyzed 77 PD patients and 77 matched healthy controls and confirmed the presence of several autoantibodies previously shown to be involved with CNS manifestations (84). The anti-dsDNA seropositive PD patients had a significantly higher prevalence of dyskinesia than their control counterparts, and similar results were observed for anti-brain lysate antibodies. In terms of non-motor symptoms, depression is one of the most common symptoms in PD patients and has a strong positive correlation with the presence of anti-dsDNA and anti-brain lysate autoantibodies. Constipation is another well-known non-motor symptom of PD, and it has been reported to occur at median frequency of 40–50%, based on the definition (bowel movement frequency <1 per day) and clinical tools used (85). Among the indicators of impaired gastrointestinal motility in PD, only constipation may precede the onset of motor symptoms and can be an independent risk factor of PD (86), which may herald PD or related synucleinopathies neurodegenerative conditions by at least 5 years (87). Constipation has been confirmed to have an intimate relation with gut microbiota disorders. Evidence has shown that a pathogenic pathway exists between PD and small intestinal bacterial overgrowth (SIBO) (88), and the prevalence of SIBO is significantly higher in PD patients than in controls (89). Meanwhile, the “microbiome-gut-brain axis disorder” theory has been proposed to explain the pathogenesis of PD, which is significantly modulated by the gut microbiota via immunological and gut bacterial antigens exposed to the immune system, which might also be autoimmunogenic (90). Dobbs et al. also proposed that gut microbiota disorders incur autoimmunity, ultimately resulting in neuronal damage and PD (91). Moreover, much research has provided new insights into the potential link between α-syn and the gut microbiota. Oueslati et al. described the appearance of α-syn-positive inclusions in the gastrointestinal track, notably in the colon, and elaborated the transmission of α-syn to the dorsal motor nucleus through the vagus nerve. This mechanism was further detailed by Braak's research showing that the α-syn pathology started in the submucosal plexus of the enteric nervous system and was propagated in a retrograde manner to the CNS (92). More specifically, α-syn aggregations reach the preganglionic cholinergic neurons of the dorsal motor nucleus and eventually reaching the cerebral cortex via the retrograde axonal and transneuronal transport. In PD rat models, the increased expression of α-syn emerges earlier in the intestinal mucosa than in the brain (93). We observed intestinal flora variance in a PD mouse model induced by rotenone (data not published) and successfully detected and labeled α-syn in the intestinal mucosa to monitor its location and abnormal aggregation. All of these findings support the hypothesis that pathological progression spreads from the gut to the brain. In α-syn transgenic mice, intestinal flora disturbances have been observed and promoted constipation and motor dysfunction compared with the normal control mice. Furthermore, intestinal flora disturbances broke the immune tolerance mechanism of Tregs, leading to the activation of autoimmunity (94, 95). Excessive stimulation of the innate immune system caused by gut dysbiosis and/or SIBO might induce systemic inflammation, further incurring the activation of enteric glial cells and contributing to the initiation of α-syn misfolding, which is required for motor deficits (96). The involvement of α-syn in the autoimmunity-associated pathogenesis in the CNS of PD has been discussed in previous parts of this article; here, we provide additional evidence that α-syn-associated autoimmunity affects various aspects of PD, including both motor and non-motor symptoms, such as constipation. However, the exact mechanism driving α-syn aggregation in autoimmunity as well as its relationship with disease progression and neuronal degeneration remains unknown (Figure 3).

## Other autoimmune diseases combined with PD

Autoimmunity can destroy the whole body, and at least 80 types of autoimmune diseases have been reported to date. There is no doubt that some of them tangle with each other and share a common pathogenesis. As early as 1999, Bonuccelli et al. reported that dyskinesias and “on-off” phenomena were abated when euthyroidism was restored in advanced PD accompanied by thyrotoxicosis (97). They further verified the possible neurological differences between PD and non-PD patients as being related to thyroid autoimmunity and function. Recently, increasing data have confirmed that hypo- and hyperthyroidism are more prevalent in PD patients than in normal controls because thyroperoxidase can influence PD nitrosative stress as well as serum α-syn nitrosylation (98). Likewise, in the last year, Bartkiewicz et al. confirmed that patients suffering bullous pemphigoid (BP), an autoimmune blistering dermatosis that occurs in the elderly, were more likely to suffer from neurological and psychiatric diseases, particularly prior to the diagnosis of BP (99). The autoantibodies bound two components of keratinocyte hemidesmosomal proteins, type XVII collagen/BPAG2 (BP180) and BPAG1 (BP230), as the autoantigens, which were also expressed in neuronal tissue. In addition, three types of autoantibodies, namely, anti-neuronal, anti-brain lysate, and anti-dsDNA antibodies, in patients with both PD and systemic lupus erythematous (SLE) were strongly associated with some clinical manifestations of PD, particularly dyskinesia and depression (100). A population-based case-control study in China last year also focused on the associations between autoimmune disease and PD (101). In this study, the overall incidence rate of PD was 30% higher in the autoimmune rheumatic disease (ARD) cohort than in the non-ARD cohort. Additional prospective studies should be conducted to confirm whether the activity and the severity of this autoimmune disease increase the risk of PD. Recent genome-wide association studies (GWAS) have tested the possible common genetic risk variants conveying risks for both PD and autoimmune diseases; 17 novel loci with overlap were identified, indicating that PD and other autoimmune diseases share genetic pathways (102). These results, from both fundamental and clinical studies, suggest that PD is closely associated with autoimmune diseases, further supporting the hypothesis that autoimmune mechanisms promote the development of PD.

Now that we have discussed this issue from a clinical perspective, immunotherapeutic strategies for PD cannot be neglected. Current treatments, including dopamine replacement therapy and alleviating the damage of oxidative stress and inflammation, seem insufficient and are limited in treating PD because most of them have only a therapeutic aim rather than both a therapeutic and prophylactic aim. The *in vivo* data presented by Zhu et al. demonstrated a significant suppression of T cell mediated autoimmunity in carbidopa (one of the classical medicines against PD) treated mice compared with untreated mice (103). It revealed that to prevent autoimmune disorder in PD would be a new target for drug development. Meanwhile other new approaches for treating PD are also urgently needed. Accumulating data have shown that intracerebral injections of recombinant human α-syn can successfully expand nTreg and iTreg populations in a dose-dependent manner, accompanied by decreased α-syn aggregation in DNs and synapses and reduced neurodegeneration (104); this approach has potential as an immune therapy for PD. The exact mechanism might be that the induced α-syn-specific antibodies neutralize the α-syn deposits and harness the neuroinflammation by modulating the microglial phagocytosis of antibody-antigen complexes (105). Meanwhile, it has been proposed that the high affinity of α-syn antibodies to their α-syn antigen allows them to neutralize the neurotoxic α-syn aggregates without interfering with beneficial monomeric α-syn (106). In summary, an impaired capacity for immune clearance and blocking toxic α-syn aggregates might play critical roles in the pathogenesis of PD. Therefore, immunotherapy with α-syn antibodies could be a new alternative approach for effectively treating PD.

## Conclusion

In all, autoimmunity disorders are one of the main mechanisms of PD pathogenesis and development and have gained increasing attention in recent years. Based on the latest research advances, including our laboratory data, conclusions can be drawn that both innate and adaptive immunity become pathogenic when self-antigen tolerance is lost. From all evidence, when facing an autoimmunity attack, the CNS cannot escape. To decipher the mechanisms of autoimmunity disorders involved in PD, the deletion or mutation of PD-related genes and the dysfunction of their encoded proteins should be studied. The involvement of α-syn provides strong evidence that this protein quite possibly acts as the first target of autoimmune attack, followed by the sustained activation of DCs and microglia, inflammation, and immune cell recruitment. Our review suggests that α-syn will no longer merely be the PD pathological hallmark but will also become one of the main targets of autoimmunity attack. In addition, NM, as another novel autoantigen released from dead DNs, is phagocytized by DCs and then induces the activation of microglia, contributing to the autoimmune aggravation of PD. Through developing neuroimmunoregulatory therapies, many new therapeutic options will become available to PD patients. These achievements will benefit both diagnostics and treatments. This review sheds light on autoimmunity associated with the etiology and pathogenesis of PD from a new perspective and further proposes some possible therapeutic targets and methods for PD (Table 1).

**Table 1:** Autoimmunity can be a cause of PD.

**Relationship**	**Research object**	**Evidence**	**References**
Genetic regulation of autoimmunity in PD	*PINK1, Parkin*	Absence of *PINK1/Parkin* leads to the mitochondrial aberrations by triggering immune system disorders (reduced immuno-surveillance or activated autoimmunity).	(34, 43–47)
	*DJ-1*	Absence of *DJ-1* leads to abnormal proliferation of nTregs and iTregs, and result in autoimmunity.	(48–50)
Pathogenic protein function in autoimmunity- associated PD	α-syn	Post-translational modifications and mutation of α-syn can be recognized as the autoantigen by the central immune system.	(56–58, 62, 64, 65)
Immune cells and autoimmunity in PD	DC	NM is an autoantigen released from dead DNs that stimulates the functional activation of DCs, triggering an autoimmune response and leading to microglial activation.	(28, 72–75)
	Microglia	Auto-aggressive loop initiated by DCs along with NM would be enhanced and amplified by microglial activation.	(78–80)
Clinical features and autoimmunity in PD	Tremor/dyskinesia/depression	Various autoantibodies have a strong positive correlation with these motor/non-motor symptoms.	(29, 83, 84)
	Constipation	Constipation is related to the gut dysbiosis and/or SIBO, which incurring the activation of enteric glial cells and contributing to the initiation of α-syn misfolding.	(90–93)
Other autoimmune diseases combined with PD	Hypothyroidism/hyperthyroidism/BP/SLE/ARD	Other autoimmune diseases may share genetic pathways with PD and are correlated closely with some clinical manifestations of PD.	(97–102)

*PD, Parkinson's disease; α-syn, α-synuclein; DC, dendritic cell; NM, neuromelanin; SIBO, small intestinal bacterial overgrowth; BP, bullous pemphigoid; SLE, systemic lupus erythematous; ARD, autoimmune rheumatic disease*.

## Author contributions

TJ and GL wrote the paper. JX supervised the figures. SG and XC supervised and wrote the paper.

### Conflict of interest statement

The authors declare that the research was conducted in the absence of any commercial or financial relationships that could be construed as a potential conflict of interest.

## Abbreviations

α-Syn: α-Synuclein
AD: Alzheimer's disease
APCs: antigen presenting cells
ARD: autoimmune rheumatic disease
BP: bullous pemphigoid
CNS: central nervous system
CSF: cerebrospinal fluid
DC: dendritic cell
DN: dopaminergic neuron
dsDNA: double-stranded deoxyribonucleic acid
GWAS: genome-wide association studies
HSV1: herpes simplex virus 1
Ig: immunoglobulin
IL: Interleukin
iTregs: induced regulatory T cells
LDLs: low-density lipoproteins
LPC: lipophosphatidylcholine
MAPK: mitogen-activated protein kinase
MDVs: mitochondria-derived vesicles
MHC: major histocompatibility complex
MitAP: mitochondrial antigens presentation
MPTP: 1-methyl-4-phenyl-1,2,3,6-tetrahydropyridine
NF-κB: nuclear factor kappaB
NM: neuromelanin
nTregs: natural regulatory T cells
PARK7: Parkinson's Disease Protein 7
PD: Parkinson's disease
PRRs: pattern recognition receptors
SIBO: small intestinal bacterial overgrowth
SLE: systemic lupus erythematous
SNpc: substantia nigra pars compacta
TNF: tumor necrosis factor
Tregs: regulatory T cells.

## References

[1] Cardenas-Roldan J Rojas-Villarraga A Anaya JM. How do autoimmune diseases cluster in families? A systematic review and meta-analysis. BMC Med. (2013) 11:73. 10.1186/1741-7015-11-73 23497011 PMC3655934

[2] Cooper GS Bynum ML Somers EC. Recent insights in the epidemiology of autoimmune diseases: improved prevalence estimates and understanding of clustering of diseases. J Autoimmun. (2009) 33:197–207. 10.1016/j.jaut.2009.09.008 19819109 PMC2783422

[3] Chastain EM Miller SD. Molecular mimicry as an inducing trigger for CNS autoimmune demyelinating disease. Immunol Rev. (2012) 245:227–38. 10.1111/j.1600-065X.2011.01076.x 22168423 PMC3586283

[4] van Schie KA Wolbink GJ Rispens T. Cross-reactive and pre-existing antibodies to therapeutic antibodies–Effects on treatment and immunogenicity. mAbs (2015) 7:662–71. 10.1080/19420862.2015.1048411 25962087 PMC4623040

[5] Qiao YC Pan YH Ling W Tian F Chen YL Zhang XX. The Yin and Yang of regulatory T cell and therapy progress in autoimmune disease. Autoimmun Rev. (2017) 16:1058–70. 10.1016/j.autrev.2017.08.001 28778708

[6] Iikuni N Lourenco EV Hahn BH La Cava A. Cutting edge: regulatory T cells directly suppress B cells in systemic lupus erythematosus. J Immunol. (2009) 183:1518–22. 10.4049/jimmunol.0901163 19570829 PMC2730469

[7] Mizoguchi A Bhan AK. A case for regulatory B cells. J Immunol. (2006) 176:705–10. 10.4049/jimmunol.176.2.705 16393950

[8] Junker A Bruck W. Autoinflammatory grey matter lesions in humans: cortical encephalitis, clinical disorders, experimental models. Curr Opin Neurol. (2012) 25:349–57. 10.1097/WCO.0b013e3283534a8a 22543404

[9] Kawai T Akira S. Innate immune recognition of viral infection. Nat Immunol. (2006) 7:131–7. 10.1038/ni1303 16424890

[10] Doecke JD Laws SM Faux NG Wilson W Burnham SC Lam CP. Blood-based protein biomarkers for diagnosis of Alzheimer disease. Arch Neurol. (2012) 69:1318–25. 10.1001/archneurol.2012.1282 22801742 PMC6287606

[11] Patejdl R Zettl UK. Spasticity in multiple sclerosis: contribution of inflammation, autoimmune mediated neuronal damage and therapeutic interventions. Autoimmun Rev. (2017) 16:925–36. 10.1016/j.autrev.2017.07.004 28698092

[12] Ray S Britschgi M Herbert C Takeda-Uchimura Y Boxer A Blennow K. Classification and prediction of clinical Alzheimer's diagnosis based on plasma signaling proteins. Nat Med. (2007) 13:1359–62. 10.1038/nm1653 17934472

[13] Goris A Pauwels I Gustavsen MW van Son B Hilven K Bos SD. Genetic variants are major determinants of CSF antibody levels in multiple sclerosis. Brain (2015) 138:632–43. 10.1093/brain/awu405 25616667 PMC4408440

[14] De Stefano N Matthews PM Fu L Narayanan S Stanley J Francis GS. Axonal damage correlates with disability in patients with relapsing-remitting multiple sclerosis. Results of a longitudinal magnetic resonance spectroscopy study. Brain (1998) 121:1469–77 9712009 10.1093/brain/121.8.1469

[15] Tallantyre EC Bo L Al-Rawashdeh O Owens T Polman CH Lowe JS. Clinico-pathological evidence that axonal loss underlies disability in progressive multiple sclerosis. Mult. Scler. (2010) 16:406–11. 10.1177/1352458510364992 20215480

[16] Agrawal A Tay J Ton S Agrawal S Gupta S. Increased reactivity of dendritic cells from aged subjects to self-antigen, the human DNA. J Immunol. (2009) 182:1138–45. 10.4049/jimmunol.182.2.1138 19124757 PMC2621318

[17] Agrawal A Tay J Yang GE Agrawal S Gupta S. Age-associated epigenetic modifications in human DNA increase its immunogenicity. Aging (2010) 2:93–100. 10.18632/aging.100121 20354270 PMC2850145

[18] Bueno V Sant'Anna OA Lord JM. Ageing and myeloid-derived suppressor cells: possible involvement in immunosenescence and age-related disease. Age (2014) 36:9729. 10.1007/s11357-014-9729-x 25399072 PMC4233024

[19] Rosato E Salsano F. Immunity, autoimmunity and autoimmune diseases in older people. J Biol Regulat Homeostat Agents (2008) 22:217. 19036223

[20] De Virgilio A Greco A Fabbrini G Inghilleri M Rizzo MI Gallo A. Parkinson's disease: autoimmunity and neuroinflammation. Autoimmun Rev. (2016) 15:1005–11. 10.1016/j.autrev.2016.07.022 27497913

[21] Holmans P Moskvina V Jones L Sharma M Vedernikov A Buchel F. A pathway-based analysis provides additional support for an immune-related genetic susceptibility to Parkinson's disease. Human Mol Genet. (2013) 22:1039–49. 10.1093/hmg/dds492 23223016 PMC3561909

[22] Kubo M Kamiya Y Nagashima R Maekawa T Eshima K Azuma S. LRRK2 is expressed in B-2 but not in B-1 B cells, and downregulated by cellular activation. J Neuroimmunol. (2010) 229:123–8. 10.1016/j.jneuroim.2010.07.021 20728949

[23] Moller A Perrild H Pedersen H Hoier-Madsen M. Parkinson's disease and autoimmunity. Acta Neurol Scand. (1989) 79:173–5. 2711825 10.1111/j.1600-0404.1989.tb03733.x

[24] Liu B Gao HM Hong JS. Parkinson's disease and exposure to infectious agents and pesticides and the occurrence of brain injuries: role of neuroinflammation. Environ Health Perspect. (2003) 111:1065–73. 10.1289/ehp.6361 12826478 PMC1241555

[25] Netea MG Joosten LA Latz E Mills KH Natoli G Stunnenberg HG. Trained immunity: a program of innate immune memory in health and disease. Science (2016) 352:aaf1098. 10.1126/science.aaf1098 27102489 PMC5087274

[26] Fiszer U Mix E Fredrikson S Kostulas V Olsson T Link H. gamma delta+ T cells are increased in patients with Parkinson's disease. J Neurol Sci. (1994) 121:39–45. 8133310 10.1016/0022-510x(94)90154-6

[27] Cebrian C Zucca FA Mauri P Steinbeck JA Studer L Scherzer CR. MHC-I expression renders catecholaminergic neurons susceptible to T-cell-mediated degeneration. Nat Commun. (2014) 5:3633. 10.1038/ncomms4633 24736453 PMC4024461

[28] Double KL Rowe DB Carew-Jones FM Hayes M Chan DK Blackie J. Anti-melanin antibodies are increased in sera in Parkinson's disease. Exp Neurol (2009) 217:297–301. 10.1016/j.expneurol.2009.03.002 19289120

[29] Papachroni KK Ninkina N Papapanagiotou A Hadjigeorgiou GM Xiromerisiou G Papadimitriou A. Autoantibodies to alpha-synuclein in inherited Parkinson's disease. J Neurochem. (2007) 101:749–56. 10.1111/j.1471-4159.2006.04365.x 17448146 PMC3154646

[30] Yanamandra K Gruden MA Casaite V Meskys R Forsgren L Morozova-Roche LA. alpha-synuclein reactive antibodies as diagnostic biomarkers in blood sera of Parkinson's disease patients. PLoS ONE (2011) 6:e18513. 10.1371/journal.pone.0018513 21541339 PMC3081826

[31] Brochard V Combadiere B Prigent A Laouar Y Perrin A Beray-Berthat V. Infiltration of CD4+ lymphocytes into the brain contributes to neurodegeneration in a mouse model of Parkinson disease. J Clin Invest. (2009) 119:182–92. 10.1172/jci36470 19104149 PMC2613467

[32] Orr CF Rowe DB Mizuno Y Mori H Halliday GM. A possible role for humoral immunity in the pathogenesis of Parkinson's disease. Brain (2005) 128:2665–74. 10.1093/brain/awh625 16219675

[33] Baba Y Kuroiwa A Uitti RJ Wszolek ZK Yamada T. Alterations of T-lymphocyte populations in Parkinson disease. Parkinsonism Relat Dis. (2005) 11:493–8. 10.1016/j.parkreldis.2005.07.005 16154792

[34] Mosley RL Hutter-Saunders JA Stone DK Gendelman HE. Inflammation and adaptive immunity in Parkinson's disease. Cold Spring Harb Persp Med (2012) 2:a009381. 10.1101/cshperspect.a009381 22315722 PMC3253034

[35] Cho JH Gregersen PK. Genomics and the multifactorial nature of human autoimmune disease. N Engl J Med. (2011) 365:1612–23. 10.1056/NEJMra1100030 22029983

[36] Koutsilieri E Lutz MB Scheller C. Autoimmunity, dendritic cells and relevance for Parkinson's disease. J Neural Trans. (2013) 120:75–81. 10.1007/s00702-012-0842-7 22699458 PMC3535404

[37] Houlden H Singleton AB. The genetics and neuropathology of Parkinson's disease. Acta Neuropathol. (2012) 124:325–38. 10.1007/s00401-012-1013-5 22806825 PMC3589971

[38] Narendra DP Jin SM Tanaka A Suen DF Gautier CA Shen J. PINK1 is selectively stabilized on impaired mitochondria to activate Parkin. PLoS Biol. (2010) 8:e1000298. 10.1371/journal.pbio.1000298 20126261 PMC2811155

[39] Pickrell AM Youle RJ. The roles of PINK1, parkin, and mitochondrial fidelity in Parkinson's disease. Neuron (2015) 85:257–73. 10.1016/j.neuron.2014.12.007 25611507 PMC4764997

[40] Qu D Hage A Don-Carolis K Huang E Joselin A Safarpour F. BAG2 Gene-mediated regulation of pink1 protein is critical for mitochondrial translocation of parkin and neuronal survival. J Biol Chem. (2015) 290:30441–52. 10.1074/jbc.M115.677815 26538564 PMC4683266

[41] Heo JM Ordureau A Paulo JA Rinehart J Harper JW. The PINK1-PARKIN mitochondrial ubiquitylation pathway drives a program of OPTN/NDP52 recruitment and TBK1 activation to promote mitophagy. Mol Cell (2015) 60:7–20. 10.1016/j.molcel.2015.08.016 26365381 PMC4592482

[42] Lazarou M Sliter DA Kane LA Sarraf SA Wang C Burman JL. The ubiquitin kinase PINK1 recruits autophagy receptors to induce mitophagy. Nature (2015) 524:309–14. 10.1038/nature14893 26266977 PMC5018156

[43] Ellis GI Zhi L Akundi R Bueler H Marti F. Mitochondrial and cytosolic roles of PINK1 shape induced regulatory T-cell development and function. Eur J Immunol. (2013) 43:3355–60. 10.1002/eji.201343571 24037540 PMC4539263

[44] Mouton-Liger F Jacoupy M Corvol JC Corti O. PINK1/Parkin-Dependent mitochondrial surveillance: from pleiotropy to parkinson's disease. Front Mol Neurosci. (2017) 10:120. 10.3389/fnmol.2017.00120 28507507 PMC5410576

[45] Matheoud D Sugiura A Bellemare-Pelletier A Laplante A Rondeau C Chemali M. Parkinson's disease-related proteins pink1 and parkin repress mitochondrial antigen presentation. Cell (2016) 166:314–27. 10.1016/j.cell.2016.05.039 27345367

[46] Kannarkat GT Boss JM Tansey MG. The role of innate and adaptive immunity in Parkinson's disease. J Parkinson's Dis. (2013) 3:493–514. 10.3233/jpd-130250 24275605 PMC4102262

[47] Louveau A Smirnov I Keyes TJ Eccles JD Rouhani SJ Peske JD. Structural and functional features of central nervous system lymphatic vessels. Nature (2015) 523:337–41. 10.1038/nature14432 26030524 PMC4506234

[48] Bettelli E Carrier Y Gao W Korn T Strom TB Oukka M. Reciprocal developmental pathways for the generation of pathogenic effector TH17 and regulatory T cells. Nature (2006) 441:235–8. 10.1038/nature04753 16648838

[49] Sakaguchi S. Naturally arising Foxp3-expressing CD25+CD4+ regulatory T cells in immunological tolerance to self and non-self. Nat Immunol. (2005) 6:345–52. 10.1038/ni1178 15785760

[50] Singh Y Chen H Zhou Y Foller M Mak TW Salker MS. Differential effect of DJ-1/PARK7 on development of natural and induced regulatory T cells. Sci Rep. (2015) 5:17723. 10.1038/srep17723 26634899 PMC4669505

[51] Trempe JF Fon E. Structure and Function of Parkin, PINK1, and DJ-1, the three musketeers of neuroprotection. Front Neurol. (2013) 4:38. 10.3389/fneur.2013.00038 23626584 PMC3630392

[52] Wang G Pan J Chen SD. Kinases and kinase signaling pathways: potential therapeutic targets in Parkinson's disease. Progress Neurobiol. (2012) 98:207–21. 10.1016/j.pneurobio.2012.06.003 22709943

[53] Yang H Zhou HY Li B Niu GZ Chen SD. Downregulation of parkin damages antioxidant defenses and enhances proteasome inhibition-induced toxicity in PC12 cells. J Neuroimmune Pharmacol. (2007) 2:276–83. 10.1007/s11481-007-9082-2 18040862

[54] Jiang T Sun Q Chen S. Oxidative stress: a major pathogenesis and potential therapeutic target of antioxidative agents in Parkinson's disease and Alzheimer's disease. Progress Neurobiol. (2016) 147:1–19. 10.1016/j.pneurobio.2016.07.005 27769868

[55] Jiang TF Chen SD. Dysfunction of two lysosome degradation pathways of alpha-synuclein in Parkinson's disease: potential therapeutic targets? Neurosci Bull. (2012) 28:649–57. 10.1007/s12264-012-1263-1 22961477 PMC5561915

[56] Benner EJ Banerjee R Reynolds AD Sherman S Pisarev VM Tsiperson V. Nitrated alpha-synuclein immunity accelerates degeneration of nigral dopaminergic neurons. PLoS ONE (2008) 3:e1376. 10.1371/journal.pone.0001376 18167537 PMC2147051

[57] Cao S Theodore S Standaert DG. Fcgamma receptors are required for NF-kappaB signaling, microglial activation and dopaminergic neurodegeneration in an AAV-synuclein mouse model of Parkinson's disease. Mol Neurodegenerat. (2010) 5:42. 10.1186/1750-1326-5-42 20977765 PMC2975641

[58] Allen Reish HE Standaert DG. Role of alpha-synuclein in inducing innate and adaptive immunity in Parkinson disease. J Parkinson's Dis. (2015) 5:1–19. 10.3233/jpd-140491 25588354 PMC4405142

[59] Hara S Arawaka S Sato H Machiya Y Cui C Sasaki A. Serine 129 phosphorylation of membrane-associated α-synuclein modulates dopamine transporter function in a G protein–coupled receptor kinase–dependent manner. Mol Biol Cell (2013) 24:1649–60. 10.1091/mbc.E12-12-0903 23576548 PMC3667719

[60] Shahpasandzadeh H Popova B Kleinknecht A Fraser PE Outeiro TF Braus GH. Interplay between sumoylation and phosphorylation for protection against α-synuclein inclusions. J Biol Chem. (2014) 289:31224–40. 10.1074/jbc.M114.559237 25231978 PMC4223324

[61] Huang B Wu S Wang Z Ge L Rizak JD Wu J. Phosphorylated α-Synuclein accumulations and lewy body-like pathology distributed in parkinson's disease-related brain areas of aged rhesus monkeys treated with MPTP. Neuroscience (2018) 379:302–15. 10.1016/j.neuroscience.2018.03.026 29592843

[62] Anderson JP Walker DE Goldstein JM de Laat R Banducci K Caccavello RJ. Phosphorylation of Ser-129 is the dominant pathological modification of alpha-synuclein in familial and sporadic Lewy body disease. J Biol Chem. (2006) 281:29739–52. 10.1074/jbc.M600933200 16847063

[63] Games D Seubert P Rockenstein E Patrick C Trejo M Ubhi K. Axonopathy in an alpha-synuclein transgenic model of Lewy body disease is associated with extensive accumulation of C-terminal-truncated alpha-synuclein. Am. J Pathol. (2013) 182:940–53. 10.1016/j.ajpath.2012.11.018 23313024 PMC3589076

[64] Li W West N Colla E Pletnikova O Troncoso JC Marsh L. Aggregation promoting C-terminal truncation of alpha-synuclein is a normal cellular process and is enhanced by the familial Parkinson's disease-linked mutations. Proc Nat Acad Sci USA. (2005) 102:2162–7. 10.1073/pnas.0406976102 15684072 PMC548541

[65] Caggiu E Paulus K Arru G Piredda R Sechi GP Sechi LA. Humoral cross reactivity between alpha-synuclein and herpes simplex-1 epitope in Parkinson's disease, a triggering role in the disease? J Neuroimmunol. (2016) 291:110–4. 10.1016/j.jneuroim.2016.01.007 26857504

[66] Bandres-Ciga S Cookson MR. Alpha-synuclein triggers T-cell response. Is Parkinson's disease an autoimmune disorder? Mov Dis. (2017) 32:1327. 10.1002/mds.27116 28782845

[67] Wlodarczyk A Lobner M Cedile O Owens T. Comparison of microglia and infiltrating CD11c(+) cells as antigen presenting cells for T cell proliferation and cytokine response. J Neuroinflammation (2014) 11:57. 10.1186/1742-2094-11-57 24666681 PMC3987647

[68] Bigley V Barge D Collin M. Dendritic cell analysis in primary immunodeficiency. Curr Opin Allergy Clin Immunol. (2016) 16:530–40. 10.1097/aci.0000000000000322 27755182 PMC5087571

[69] Thomson AW Robbins PD. Tolerogenic dendritic cells for autoimmune disease and transplantation. Ann Rheum Dis. (2008) 67 (Suppl. 3):iii90–6. 10.1136/ard.2008.099176 19022823

[70] Agrawal A Sridharan A Prakash S Agrawal H. Dendritic cells and aging: consequences for autoimmunity. Expert Rev Clin Immunol. (2012) 8:73–80. 10.1586/eci.11.77 22149342 PMC3285507

[71] Platt AM Randolph GJ. Does deleting dendritic cells delete autoimmunity? Immunity (2010) 33:840–2. 10.1016/j.immuni.2010.12.003 21168776 PMC4031752

[72] Ito S Yamanaka Y Ojika M Wakamatsu K. The metabolic fate of ortho-quinones derived from catecholamine metabolites. Int J Mol Sci. (2016) 17:164. 10.3390/ijms17020164 26828480 PMC4783898

[73] Haining R Achat-Mendes C. Neuromelanin, one of the most overlooked molecules in modern medicine, is not a spectator. Neural Regenerat Res. (2017) 12:372–5. 10.4103/1673-5374.202928 28469642 PMC5399705

[74] Oberlander U Pletinckx K Dohler A Muller N Lutz MB Arzberger T. Neuromelanin is an immune stimulator for dendritic cells in vitro. BMC Neurosci. (2011) 12:116. 10.1186/1471-2202-12-116 22085464 PMC3225309

[75] Depboylu C Schafer MK Arias-Carrion O Oertel WH Weihe E Hoglinger GU. Possible involvement of complement factor C1q in the clearance of extracellular neuromelanin from the substantia nigra in Parkinson disease. J Neuropathol Exp Neurol. (2011) 70:125–32. 10.1097/NEN.0b013e31820805b9 21343881

[76] Bailey SL Schreiner B McMahon EJ Miller SD. CNS myeloid DCs presenting endogenous myelin peptides 'preferentially' polarize CD4+ T(H)-17 cells in relapsing EAE. Nat Immunol. (2007) 8:172–80. 10.1038/ni1430 17206145

[77] Zozulya AL Ortler S Lee J Weidenfeller C Sandor M Wiendl H. Intracerebral dendritic cells critically modulate encephalitogenic versus regulatory immune responses in the CNS. J Neurosci. (2009) 29:140–52. 10.1523/jneurosci.2199-08.2009 19129392 PMC2942091

[78] Thompson KK Tsirka SE. The diverse roles of microglia in the neurodegenerative aspects of central nervous system (CNS) autoimmunity. Int J Mol Sci. (2017) 18:504. 10.3390/ijms18030504 28245617 PMC5372520

[79] Wilms H Rosenstiel P Sievers J Deuschl G Zecca L Lucius R. Activation of microglia by human neuromelanin is NF-kappaB dependent and involves p38 mitogen-activated protein kinase: implications for Parkinson's disease. FASEB J. (2003) 17:500–2. 10.1096/fj.020314fje 12631585

[80] Zhang W Phillips K Wielgus AR Liu J Albertini A Zucca FA. Neuromelanin activates microglia and induces degeneration of dopaminergic neurons: implications for progression of Parkinson's disease. Neurotox Res. (2011) 19:63–72. 10.1007/s12640-009-9140-z 19957214 PMC3603276

[81] Liu GD Ding JQ Xiao Q Chen SD. P2Y6 receptor and immunoinflammation. Neurosci Bull. (2009) 25:161–4. 10.1007/s12264009-0120-3 19448690 PMC5552562

[82] Thenganatt MA Jankovic J. Parkinson disease subtypes. JAMA Neurol. (2014) 71:499–504. 10.1001/jamaneurol.2013.6233 24514863

[83] Zappia M Crescibene L Bosco D Arabia G Nicoletti G Bagala A. Anti-GM1 ganglioside antibodies in Parkinson's disease. Acta Neurol Scand. (2002) 106:54–7. 10.1034/j.1600-0404.2002.01240.x 12067330

[84] Benkler M Agmon-Levin N Hassin-Baer S Cohen OS Ortega-Hernandez OD Levy A. Immunology, autoimmunity, and autoantibodies in Parkinson's disease. Clin Rev Allergy Immunol. (2012) 42:164–71. 10.1007/s12016-010-8242-y 21234712

[85] Knudsen K Krogh K Ostergaard K Borghammer P. Constipation in parkinson's disease: subjective symptoms, objective markers, and new perspectives. Movement Dis. (2017) 32:94–105. 10.1002/mds.26866 27873359

[86] Jost WH. Gastrointestinal dysfunction in Parkinson's Disease. J Neurol Sci. (2010) 289:69–73. 10.1016/j.jns.2009.08.020 19717168

[87] Goldman JG Postuma R. Premotor and nonmotor features of Parkinson's disease. Curr Opin Neurol. (2014) 27:434–41. 10.1097/wco.0000000000000112 24978368 PMC4181670

[88] Savica R Carlin JM Grossardt BR Bower JH Ahlskog JE Maraganore DM. Medical records documentation of constipation preceding Parkinson disease: a case-control study. Neurology (2009) 73:1752–8. 10.1212/WNL.0b013e3181c34af5 19933976 PMC2788809

[89] Fasano A Bove F Gabrielli M Petracca M Zocco MA Ragazzoni E. The role of small intestinal bacterial overgrowth in Parkinson's disease. Mov Dis. (2013) 28:1241–9. 10.1002/mds.25522 23712625

[90] Negi S Singh H Mukhopadhyay A. Gut bacterial peptides with autoimmunity potential as environmental trigger for late onset complex diseases: in-silico study. PLoS ONE (2017) 12:e0180518. 10.1371/journal.pone.0180518 28678867 PMC5498033

[91] Dobbs SM Dobbs RJ Weller C Charlett A. Link between Helicobacter pylori infection and idiopathic parkinsonism. Med Hypotheses (2000) 55:93–8. 10.1054/mehy.2000.1110 10904422

[92] Braak H de Vos RA Bohl J Del Tredici K. Gastric alpha-synuclein immunoreactive inclusions in Meissner's and Auerbach's plexuses in cases staged for Parkinson's disease-related brain pathology. Neurosci Lett. (2006) 396:67–72. 10.1016/j.neulet.2005.11.012 16330147

[93] Oueslati A Ximerakis M Vekrellis K. Protein Transmission, Seeding and degradation: key steps for alpha-synuclein prion-like propagation. Exp Neurobiol. (2014) 23:324–36. 10.5607/en.2014.23.4.324 25548532 PMC4276803

[94] Cebula A Seweryn M Rempala GA Pabla SS McIndoe RA Denning TL. Thymus-derived regulatory T cells control tolerance to commensal microbiota. Nature (2013) 497:258–62. 10.1038/nature12079 23624374 PMC3711137

[95] Sampson TR Debelius JW Thron T Janssen S Shastri GG Ilhan ZE. Gut microbiota regulate motor deficits and neuroinflammation in a model of parkinson's disease. Cell (2016) 167:1469–80.e12. 10.1016/j.cell.2016.11.018 27912057 PMC5718049

[96] Mulak A Bonaz B. Brain-gut-microbiota axis in Parkinson's disease. World J Gastroenterol. (2015) 21:10609–20. 10.3748/wjg.v21.i37.10609 26457021 PMC4588083

[97] Bonuccelli U D'Avino C Caraccio N Del Guerra P Casolaro A Pavese N. Thyroid function and autoimmunity in Parkinson's disease: a study of 101 patients. Parkinsonism Relat Dis. (1999) 5:49–53. 18591119 10.1016/s1353-8020(99)00010-3

[98] Fernandez E Garcia-Moreno JM Martin de Pablos A Chacon J. May the thyroid gland and thyroperoxidase participate in nitrosylation of serum proteins and sporadic Parkinson's disease? Antioxid Redox Signal. (2014) 21:2143–8. 10.1089/ars.2014.6072 25125346 PMC4215330

[99] Bartkiewicz P Gornowicz-Porowska J Pietkiewicz PP Swirkowicz A Bowszyc-Dmochowska M Dmochowski M. Neurodegenerative disorders, bullous pemphigoid and psoriasis: a comparative study in ethnic Poles indicates that Parkinson's disease is more relevant to bullous pemphigoid. Postepy Dermatol Alergol. (2017) 34:42–6. 10.5114/ada.2017.65619 28261030 PMC5329105

[100] Liu FC Huang WY Lin TY Shen CH Chou YC Lin CL. Inverse association of parkinson disease with systemic lupus erythematosus: a nationwide population-based study. Medicine (2015) 94:e2097. 10.1097/md.0000000000002097 26579824 PMC4652833

[101] Chang CC Lin TM Chang YS Chen WS Sheu JJ Chen YH. Autoimmune rheumatic diseases and the risk of Parkinson disease: a nationwide population-based cohort study in Taiwan. Ann Med. (2018) 50:83–90. 10.1080/07853890.2017.1412088 29224375

[102] Witoelar A Jansen IE Wang Y Desikan RS Gibbs JR Blauwendraat C. Genome-wide pleiotropy between parkinson disease and autoimmune diseases. JAMA Neurolo. (2017) 74:780–92. 10.1001/jamaneurol.2017.0469 28586827 PMC5710535

[103] Zhu H Lemos H Bhatt B Islam BN Singh A Gurav A. Carbidopa, a drug in use for management of Parkinson disease inhibits T cell activation and autoimmunity. PLoS ONE (2017) 12:e0183484. 10.1371/journal.pone.0183484 28898256 PMC5595290

[104] Christiansen JR Olesen MN Otzen DE Romero-Ramos M Sanchez-Guajardo V. Alpha-Synuclein vaccination modulates regulatory T cell activation and microglia in the absence of brain pathology. J Neuroinflam. (2016) 13:74. 10.1186/s12974-016-0532-8 27055651 PMC4825077

[105] Brudek T Winge K Folke J Christensen S Fog K Pakkenberg B. Autoimmune antibody decline in Parkinson's disease and Multiple System Atrophy; a step towards immunotherapeutic strategies. Mol Neurodegenerat. (2017) 12:44. 10.1186/s13024-017-0187-7 28592329 PMC5463400

[106] Emadi S Barkhordarian H Wang MS Schulz P Sierks MR. Isolation of a human single chain antibody fragment against oligomeric α-synuclein that inhibits aggregation and prevents α-synuclein induced toxicity. J Mol Biol. (2007) 368:1132–44. 10.1016/j.jmb.2007.02.089 17391701 PMC2235820

