# Corrigendum: The Challenge of the Pathogenesis of Parkinson's Disease: Is Autoimmunity the Culprit?

**DOI:** 10.3389/fimmu.2019.02242

**Published:** 2019-10-16

**Authors:** Tianfang Jiang, Gen Li, Jun Xu, Shane Gao, Xu Chen

**Affiliations:** ^1^Department of Neurology, Shanghai Eighth People's Hospital Affiliated to Jiang Su University, Shanghai, China; ^2^Department of Neurology & Institute of Neurology, Rui Jin Hospital Affiliated to Shanghai Jiao Tong University School of Medicine, Shanghai, China; ^3^East Hospital, Tong Ji University School of Medicine, Shanghai, China

**Keywords:** autoimmunity, Parkinson's disease, α-synuclein, autoimmune diseases, neuroimmunology

In the original article, there was a mistake in Table 1 as published. The reference “Singh Y, Chen H, Zhou Y, Foller M, Mak TW, Salker MS, et al. Differential effect of DJ-1/PARK7 on development of natural and induced regulatory T cells. *Sci Rep*. (2015) 5:17723. doi: 10.1038/srep17723” should be inserted as reference 50. The corrected Table 1 appears below.

**Table 1:** Autoimmunity can be a cause of PD.

**Relationship**	**Research object**	**Evidence**	**References**
Genetic regulation of autoimmunity in PD	*PINK1, Parkin*	Absence of *PINK1/Parkin* leads to the mitochondrial aberrations by triggering immune system disorders (reduced immuno-surveillance or activated autoimmunity).	(34, 43–47)
	*DJ-1*	Absence of *DJ-1* leads to abnormal proliferation of nTregs and iTregs, and result in autoimmunity.	(48–50)
Pathogenic protein function in autoimmunity- associated PD	α-syn	Post-translational modifications and mutation of α-syn can be recognized as the autoantigen by the central immune system.	(56–58, 62, 64, 65)
Immune cells and autoimmunity in PD	DC	NM is an autoantigen released from dead DNs that stimulates the functional activation of DCs, triggering an autoimmune response and leading to microglial activation.	(28, 72–75)
	Microglia	Auto-aggressive loop initiated by DCs along with NM would be enhanced and amplified by microglial activation.	(78–80)
Clinical features and autoimmunity in PD	Tremor/dyskinesia/depression	Various autoantibodies have a strong positive correlation with these motor/non-motor symptoms.	(29, 83, 84)
	Constipation	Constipation is related to the gut dysbiosis and/or SIBO, which incurring the activation of enteric glial cells and contributing to the initiation of α-syn misfolding.	(90–93)
Other autoimmune diseases combined with PD	Hypothyroidism/hyperthyroidism/BP/SLE/ARD	Other autoimmune diseases may share genetic pathways with PD and are correlated closely with some clinical manifestations of PD.	(97–102)

*PD, Parkinson's disease; α-syn, α-synuclein; DC, dendritic cell; NM, neuromelanin; SIBO, small intestinal bacterial overgrowth; BP, bullous pemphigoid; SLE, systemic lupus erythematous; ARD, autoimmune rheumatic disease*.

In addition, “Singh Y, Chen H, Zhou Y, Foller M, Mak TW, Salker MS, et al. Differential effect of DJ-1/PARK7 on development of natural and induced regulatory T cells. *Sci Rep*. (2015) 5:17723. doi: 10.1038/srep17723” was not cited in the article. The citation has now been inserted in the section ***Genetic Regulation of Autoimmunity in PD***, paragraph two:

“In addition to these observations, *DJ-1* (Parkinson's disease protein 7, *PARK7*) has also been reported to affect the development of natural Tregs (nTregs) and induced Tregs (iTregs, previously known as suppressor T cells). Mature Tregs with normal function, which modulate not only adaptive immunity but also innate immunity, are pivotal for maintaining thymic function, peripheral immune self-tolerance and immune system homeostasis. nTregs are generated in the thymus, while iTregs are derived from naïve CD4^+^ T cells encountering antigens in the peripheral organs. Both cell types are generally immunosuppressive through the suppression or downregulation of effector T cell proliferation (48). Their “self-check” function successfully prevents excessive effector cell reactions. On the other hand, the abnormal proliferation of both types of Tregs leads to the failure of self-/non-self-discrimination, resulting in autoimmune disease (49). Evidence reported by Singh et al. has demonstrated that *DJ-1*, one of the most classical key players responsible for PD pathogenesis, is strongly linked with neuroimmunology and multiple autoimmune responses in PD (50). In addition, *DJ-1*-deficient animal models have shown compromised iTreg induction, cell cycle progression, and cell survival and proliferation. *DJ-1*^−/−^ iTregs are more proliferative, more susceptible to cell death signals and deficient in cell division compared with wild type counterparts, as analyzed by flow cytometry and Western blotting.”

The authors apologize for this error and state that this does not change the scientific conclusions of the article in any way. The original article has been updated.
